# Heparanase expression is a prognostic indicator for postoperative survival in pancreatic adenocarcinoma

**DOI:** 10.1038/sj.bjc.6600232

**Published:** 2002-04-22

**Authors:** J Rohloff, J Zinke, K Schoppmeyer, A Tannapfel, H Witzigmann, J Mössner, C Wittekind, K Caca

**Affiliations:** Department of Medicine II, Leipzig University, Philipp-Rosenthal-Str. 27, 04103 Leipzig, Germany; Institute of Pathology, Leipzig University, Liebigstr. 26, 04103 Leipzig, Germany; Department of Surgery II, Leipzig University, Liebigstr. 20a, 04103 Leipzig, Germany

**Keywords:** hepararanase, pancreatic ductal adenocarcinoma, postoperative survival

## Abstract

Pancreatic ductal adenocarcinoma has a median survival of less than 6 months from diagnosis. This is due to the difficulty in early diagnosis, the aggressive biological behaviour of the tumour and a lack of effective therapies for advanced disease. Mammalian heparanase is a heparan-sulphate proteoglycan cleaving enzyme. It helps to degrade the extracellular matrix and basement membranes and is involved in angiogenesis. Degradation of extracellular matrix and basement membranes as well as angiogenesis are key conditions for tumour cell spreading. Therefore, we have analysed the expression of heparanase in human pancreatic cancer tissue and cell lines. Heparanase is expressed in cell lines derived from primary tumours as well as from metastatic sites. By immunohistochemical analysis, it is preferentially expressed at the invading edge of a tumour at both metastatic and primary tumour sites. There is a trend towards heparanase expression in metastasising tumours as compared to locally growing tumours. Postoperative survival correlates inversely with heparanase expression of the tumour reflected by a median survival of 34 and 17 month for heparanase negative and positive tumours, respectively. Our results suggest, that heparanase promotes cancer cell invasion in pancreatic carcinoma and could be used as a prognostic indicator for postoperative survival of patients.

*British Journal of Cancer* (2002) **86**, 1270–1275. DOI: 10.1038/sj/bjc/6600232 www.bjcancer.com

© 2002 Cancer Research UK

## 

PDA has a poor prognosis due to its aggressive biological behaviour. In the majority of cases, diagnosis is only established late in the course of the disease, when local invasive growth and distant metastasis have already occurred (Baumel *et al*, 1994; Murr *et al*, 1994; Robert Koch Institut, 2002). At that point, the opportunity for curative therapy, which is surgery at an early stage, has already passed. Even though complete resection may still be achieved, the rate of relapses is high (Connolly *et al*, 1987; Trede *et al*, 1990; Cameron *et al*, 1991). Adjuvant combination therapy may offer a small survival advantage but is associated with treatment related morbidity. It therefore is not unequivocally accepted (Kalser and Ellenberg, 1985; Ahlgren, 1996; Jeekel, 1997; Klinkenbijl *et al*, 1999). The cytotoxic agent gemcitabine is the only drug currently known to be effective for palliation. Nevertheless, the absolute gain in survival is marginal (Burris *et al*, 1997). A number of markers have been associated with therapy-resistant tumours or shorter survival. Their validity as prognostic criterion remains to be confirmed, though (Compton *et al*, 1997).

On the molecular level, several events including proto-oncogene mutations such as *K-ras* (Almoguera *et al*, 1988), tumour suppressor gene mutations in the *p16INK4a, DPC4*, and *p53* genes (Berrozpe *et al*, 1994; Caldas *et al*, 1994; Redston *et al*, 1994; Schutte *et al*, 1997; Goggins *et al*, 1998), and overexpression of a variety of growth factors and their receptors (Korc, 1998) have been described in the carcinogenesis of pancreatic cancer. The underlying causes for the aggressiveness of this cancer are still not fully understood.

Invasion of tumour cells into the surrounding tissue requires loosening of cell–cell adhesion, invasion of the basement membrane and disassembly of the extracellular matrix. Chief components of basement membrane (BM) and extracellular matrix (ECM) such as collagens, fibronectin and laminin are substrates of metalloproteinases and cysteine and serine proteases, which are known to be upregulated in metastatic cancers (Chambers and Matrisian, 1997).

Recently, another ECM degrading enzyme, mammalian heparanase, has been cloned from human placenta tissue and platelets and a putative 65 kDa precursor and a 50 kDa recombinant active enzyme have been expressed. Preliminary results suggest that heparanase may be a cell membrane protein (Hulett *et al*, 1999; Vlodavsky *et al*, 1999). The enzyme cleaves another key component of BM and ECM, namely heparan sulphate proteoglycans (HSPG) which belong to the glycosaminoglycans. Heparanase expression and function may be regulated at the transcriptional and posttranscriptional level (Gilat *et al*, 1995; Andela *et al*, 2000), though this topic awaits further clarification.

In addition to the enzymatic degradation of the ECM heparanase acts by releasing growth factors such as bFGF, heparan sulphate (HS) fragments and other enzymes such as lipoprotein lipase from the ECM. Consistent with its molecular capabilities heparanase has been shown to stimulate wound healing and tumour angiogenesis *in vivo* (Elkin *et al*, 2001). Its expression is upregulated in a number of metastatic tumour cell lines and transfection of tumour cells with heparanase cDNA conferred a highly metastatic phenotype (Hulett *et al*, 1999; Vlodavsky *et al*, 1999).

Heparanase expression has been studied in human primary tumours and tumour cell lines. Its expression correlates with tumour progression and invasiveness in colorectal and hepatocellular carcinoma (Friedmann *et al*, 2000; El-Assal *et al*, 2001). In this study, we examined the expression of heparanase protein and mRNA in human PDA cell lines and tumours. In addition, we studied the correlation of heparanase expression and stage of disease with postoperative survival in the intent to identify putative prognostic markers.

## MATERIALS AND METHODS

### Cell Culture and DNA transfection

Eight pancreatic cancer cell lines (AsPC-1, BxPC-3, Capan-1, Hs 766T, PANC-1, HPAF-II, CFPAC, MIA PaCa-2), the colorectal cancer cell line HT29 and the cervical cancer cell line HeLa were obtained from American Type Culture Collection (Manassas, USA) and maintained in the recommended growth conditions. Transfection for overexpression of Heparanase in HeLa cells was performed on cells growing at ∼70% confluence in T75 plates, using full-length Heparanase cDNA in pcDNA3 (Vlodavsky *et al*, 1999) and FuGENE 6 transfection reagent (Roche, Germany) according to the manufacturer's instruction.

### RNA-isolation and semiquantitative RT–PCR

RNA was isolated from cell lines with the RNeasy Kit (Qiagen, Germany) according to the manufacturer's instructions. After reverse transcription of 5 μg total RNA by oligo(dT) priming (Superscript, Invitrogen, USA), the resulting cDNA was amplified by PCR using Taq DNA polymerase (Qiagen, Germany) and the heparanase primers 5′-GTGATGAGGCAAGTATTCTTTGG and 5′-TC AGATGCAAGCAGCAACTTTG. The PCR conditions were an initial denaturation of 5 min at 94°C and subsequent denaturation for 30 s at 94°C, annealing for 30 s at 58°C and extension for 1 min at 72°C (26 cycles). Standard β-actin primers and identical PCR conditions were used as internal input controls. PCR products were separated by 1.5% agarose gel electrophoresis and visualised by SYBR Green staining (Biozym Diagnostik, Germany).

### Western blot analysis

Cells were dissolved in lysis buffer containing 5 M NaCl, 10% NP-40 (Igepal CA-630, Sigma-Aldrich, Germany), 1 M TRIS pH 8 and protease inhibitors (Complete Mini, EDTA free, Roche, Germany) at 4°C for 45 min and centrifuged at 13 000 r.p.m. for 20 min. The protein concentration of the supernatant was measured by the Bradford assay (BioRad, USA). 50 μg of each protein was separated by electrophoresis on 10% SDS/polyacrylamide gels and transferred to Hybond-P-Membrane (Amersham Pharmacia Biotech, UK) by semidry electroblotting (Transblot, BioRad, USA). Western blot analysis was carried out with a mouse monoclonal anti-heparanase antibody as previously described (Vlodavsky *et al*, 1999). A monoclonal antibody against β-actin (Sigma-Aldrich, USA) was used to verify equal loading of western blots. The secondary antibody used was a horseradish-peroxidase-conjugated goat-anti-mouse antibody (Pierce, USA). Immunoreactive bands were detected by enhanced chemiluminescence (SuperSignal, Pierce, USA) and exposure in a luminescent image analyser (LAS-1000plus, FujiFilm, USA).

### Patients and tissue samples

Tissue specimens were chosen from 25 patients with chronic pancreatitis, from 50 patients with ductal adenocarcinoma of the pancreas undergoing surgery of curative intent between 1995 and 1999 and from five cases with normal pancreatic tissue. The PDA patients included 26 patients, in whom the tumours were R0 resected. Each tumour was re-evaluated with regard to typing, staging and grading. Tumour typing and staging were performed using WHO (Klöppel *et al*, 2000) and UICC (Sobin and Wittekind, 1997) criteria.

### Immunohistochemistry

2–4 μm sections of formaline-fixed and paraffin-embedded specimens were deparaffinised and rehydrated. Endogenous peroxidase was blocked by 3% H_2_O_2_. Tissue was then demethylated in a microwave oven, incubated with citrate buffer, and blocked with goat serum. Sections were incubated with the monoclonal anti human heparanase antibody (Vlodavsky *et al*, 1999) or with non-immune mouse serum for negative control, respectively. Following washes with PBS sections were incubated with biotinylated goat anti-mouse IgG+IgM antibodies (BioGenex, USA), rinsed with PBS and incubated with peroxidase-labelled streptavidin. Colour was developed using Sigma Fast 3,3′-Diaminobenzidine (Sigma-Aldrich, USA). Counterstaining was performed with Mayer's hematoxylin. Samples were evaluated by microscopy and semiquantitavely analysed for heparanase expression (intense, moderate or absent staining).

### Riboprobe preparation and *in situ* hybridisation

A 482 bp fragment of the human heparanase cDNA (primers identical to RT–PCR primers) was subcloned into the pGEMT vector multiple cloning site (Promega, USA). The linearized vector was used as a template for *in vitro* transcription and digoxigenin labeling of antisense or sense riboprobe using SP6 and T7-RNA polymerase (Roche, Germany). *In situ* hybridisation was performed as described before (Vlodavsky *et al*, 1999; Friedmann *et al*, 2000). Briefly, 5 μm sections were dewaxed and rehydrated, denaturated with 0.2 N HCl for 10 min and then digested with proteinase K (200 μg ml^−1^) at 37°C for 30 min. Digestion was stopped with two changes of H_2_O. Slides were prehybridised and hybridised as described (Vlodavsky *et al*, 1999; Friedmann *et al*, 2000). Probe concentration was 2 μg ml^−1^. Washes after hybridisation, incubation with anti-digoxigenin antibodies, and calorimetric detection were performed as described before (Vlodavsky *et al*, 1999; Friedmann *et al*, 2000). Some of the slides were counterstained with Mayer's hematoxylin.

### Statistical analysis

Fisher's exact test was used to calculate the significance levels of contingency tables for differences of Heparanse expression regarding UICC stage, lymph node metastasis, distant metastasis or grading. For patients with R0 resected tumours postoperative survival periods were computed by the Kaplan–Meier method and compared by the Log Rank test. A *P*-value <0.05 was considered significant. SPSS-statistical software (SPSS), version 10, was used for data analysis.

## RESULTS

### Heparanase expression in human pancreatic cancer cell lines

**Figure 1:**
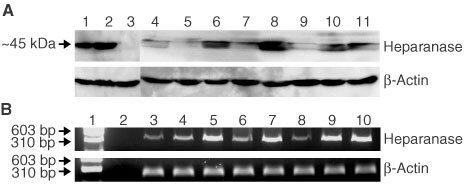
(**A**) Western blot analysis of heparanase expression in the human pancreatic cancer cell lines (AsPC-1 [4], BxPC-3 [5], Capan-1 [6], CFPAC [7], HPAF-II [8], Hs 766T [9], MIA PaCa-2 [10] and PANC-1 [11]). The colorectal cancer cell line HT-29 [1] and the cervical cancer cell line HeLa transfected with heparanase cDNA were used as positive controls. Untransfected HeLa cells were used as negative control [3]. β-Actin Western blot to confirm equal protein loading. (**B**) Semiquantitative RT–PCR of heparanase mRNA in human pancreatic cancer cell lines. DNA ladder [1], water control [2], AsPC-1 [3], BxPC-3 [4], Capan-1 [5], CFPAC [6], HPAF-II [7], Hs 766T [8], MIA PaCa-2 [9] and PANC-1 [10].

We examined eight human pancreatic cancer cell lines from different tissues of origin for the expression of heparanase mRNA and protein. Four of the cell lines (CFPAC, AsPC-1, HPAF-II, Hs 766T) had been established from distant metastases (liver, ascites) and lymph node metastases, respectively. Heparanase was expressed in all cell lines, but could hardly be detected in two cell lines (BxPC-3, Hs 766T) as determined by Western blot (Figure 1A

). The level of expression varied widely. Apparently, heparanase expression did not correlate with whether or not the cell line was derived from a metastatic site. We also looked for levels of mRNA expression by quantitative RT–PCR. Levels of mRNA correlated closely with protein expression in the tumour cell lines examined (Figure 1B).

### Heparanase expression in human pancreatic cancer tissue

**Figure 2:**
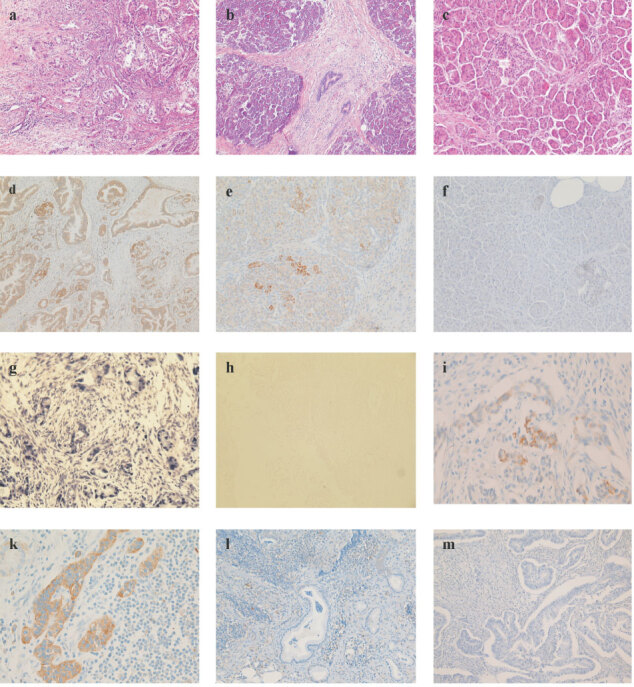
Heparanase expression and localisation in primary human pancreatic cancers (**A,D,G**), chronic pancreatitis (**B,E**) and histologically normal pancreas (**C,F**). HE staining (**A,B,C**) for histological evaluation. Immunohistochemical staining with a monoclonal α-heparanase Ab and *in situ* hybridisation with an antisense (**G**) and sense probe (**H**, negative control). Normal pancreas with absent (**F**) and chronic pancreatitis with moderate (**E**) heparanase expression. Carcinoma tissue with strong heparanase expression (**D,G**), or absent heparanase expression (**M**). Heparanase positive tumour cells invading lymph sheets (**I**). Lymph node metastasis with strong (**K**) or absent heparanase expression (**I**).

We analysed sections of tumour tissue from 50 patients (mean age 63 years, range 41–77 years, 27 male/23 female) that were operated on for pancreatic cancer and from 25 patients with chronic pancreatitis. Of the 50 tumour patients, 26 underwent a potentially curative resection, whereas the remaining 24 had microscopic evidence of non-curative resection (R1 resection). By immunohistochemical analysis, there was no detectable heparanase expression in normal pancreatic tissue. In contrast, heparanase showed high, moderate, or no expression in 17, 22, and 11 tumour specimens, respectively. Heparanase was preferentially expressed at the invading edge of PDA, though there was also some level of expression in the surrounding inflamed tissue (Figure 2

). The level of expression was higher in primary tumours than at metastatic sites. Heparanase was shown to be moderately expressed in chronic pancreatitis. *In situ* hybridisation demonstrated, that the mRNA expression pattern followed closely that of protein expression.

There was no correlation between UICC stage of disease and heparanase expression. In contrast, there was a trend towards heparanase expression in lymph node positive *vs* negative tumours (*P*=0.064) (Table 1

**Table 1:**
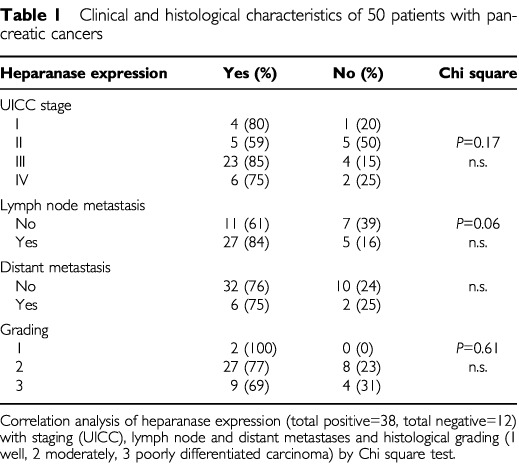
Clinical and histological characteristics of 50 patients with pancreatic cancers

and Figure 3

**Figure 3:**
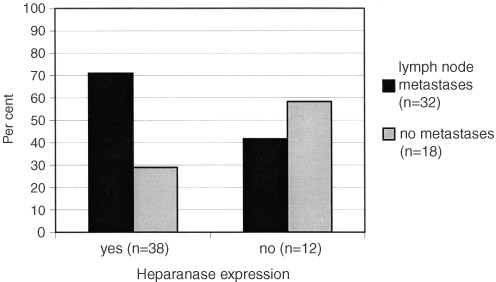
Heparanase expression in pancreatic cancers depending on lymph node metastasis (*n*=32) *vs* absent metastasis (*n*=18).

).

### Heparanase expression and postoperative survival of patients

**Figure 4:**
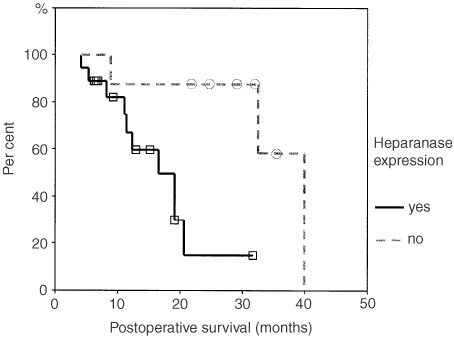
Survival curves of 26 pancreatic cancer patients with R0 resection. Kaplan–Meier plots of postoperative survival curves of patients with or without heparanase expression in the primary carcinoma. Log-rank analysis proves a shorter postoperative survival period for patients with heparanase expression in their tumours (*P*<0.01).

In the intent to determine the prognostic value of heparanase in pancreatic cancer, we have analysed heparanase expression, stage of disease and survival of those 26 patients, that had been curatively resected (R0 resection). There was a significant negative correlation between postoperative survival and heparanase expression (Figure 4

) (*P*<0.01). Mean postoperative survival was 34 months and 17 month in heparanase negative and positive tumours (R0 resection), respectively. Survival of patients with tumours expressing high or moderate levels of heparanase did not differ significantly. Multivariate analysis could not establish heparanase expression as an independent prognostic factor due to a lack of statistical power. There was no correlation between UICC stage of disease and postoperative survival in this series (data not shown).

## DISCUSSION

PDA is characterised by its local invasive growth including invasion of the perineurium and by a high frequency of metastases. In our study, heparanase was expressed in the majority of tumour specimens and expression at the invading edge of tumours could be demonstrated. This finding underscores the potential role of heparanase in tumour invasion and may partly explain the aggressiveness of PDA. We could confirm previous results that heparanase expression levels are higher at the primary tumour site than at metastatic sites (Koliopanos *et al*, 2001). This possibly reflects the need to break down physiological restraints like BM and ECM before a tumour cell can metastasise. How expression of heparanase is downregulated once a cell has settled at its metastatic site is still unresolved. In light of the results of other studies of heparanase expression in various tumours (Friedmann *et al*, 2000; El-Assal *et al*, 2001; Ginath *et al*, 2001) heparanase activation appears to be a crucial step for a tumour to become systemic.

Therapeutic options in PDA are scarce. There is no established adjuvant therapy after successfull operation despite the fact that the disease frequently relapses. Palliative treatments apart from best supportive care offer only marginal improvements in quality of life and survival, if any (Ahlgren, 1996; Burris *et al*, 1997; Jeekel, 1997). Therefore, it would be helpful to identify patients that might benefit from adjuvant therapy. We found that patients with heparanase expressing PDA had a significantly shorter postoperative survival than patients with no heparanase expression. In contrast, neither stage of disease nor lymph node involvement predicted prognosis, although this may be due to the small study size. Our finding is in agreement with previously published results (Koliopanos *et al*, 2001). We have to caution, though, that the number of patients in our study precluded a multivariate analysis. Prospective studies could lend further support to our finding. The relatively long postoperative survival periods in our study are probably due to a positive selection of patients whose tumours were deemed to be resectable preoperatively.

An important question is how heparanase expression and function are regulated under different physiological and pathophysiological conditions. Clues to physiological mechanisms may be infered from the role of heparanase in pregnancy, morphogenesis and development (Dempsey *et al*, 2000; Goldshmidt *et al*, 2001). Heparanase expression is also evident in inflammation, angiogenesis and cancer metastasis (Vlodavsky and Friedmann, 2001). Heparanase is expressed by a variety of cell types and helps the cells to cross physiological boundaries, as is the case with trophoblasts in the placenta, leukocyte extravasation, or tumour cell metastasis (Parish *et al*, 2001). There are as yet few identified molecular mechanisms of heparanase regulation. These comprise transcriptional and posttranscriptional mechanisms such as tissue-pH and regulation by NF-κB and proto-oncogenes (Schwarz *et al*, 1990; Gilat *et al*, 1995; Andela *et al*, 2000). In our study expression of heparanase mRNA and protein *in vivo* showed significant overlap. Transcriptional upregulation therefore appears to be the main way of PDA cells to benefit from the prometastatic and angiogenic effects of heparanase.

The key role of heparanase in tumorigenesis and the existing evidence for only one endogenous mammalian heparan sulphate degrading endoglycosidase (Hulett *et al*, 1999; Vlodavsky *et al*, 1999) has spurred efforts to develop therapeutic applications. So far, non-anticoagulant heparins and sulphated oligosaccharides show promising results in animal studies of tumour spreading (Miao *et al*, 1999; Parish *et al*, 1999). A drug of the latter group, Phosphomannopentaose sulfate (PI-88), slowed tumour growth and metastasis and reduced the vascularity of tumours both *in vitro* and *in vivo* in animals (Parish *et al*, 1999). Hopefully, this and other studies will emerge into clinical trials of the most promising of these drugs.
